# Comprehensive profiling of chemokine and NETosis-associated genes in sarcopenia: construction of a machine learning-based diagnostic nomogram

**DOI:** 10.3389/fmed.2025.1606430

**Published:** 2025-06-23

**Authors:** Yingwei Wang, Le Wang, Yan Zhang, Minghui Wang, Huaying Zhao, Cheng Huang, Huaiyang Cai, Shuangyang Mo

**Affiliations:** ^1^Clinical Nutrition/Gastroenterology Department, Liuzhou People’s Hospital Affiliated to Guangxi Medical University, Liuzhou, China; ^2^Department of Gastroenterology, The First Affiliated Hospital of Shandong First Medical University and Shandong Provincial Qianfoshan Hospital, Jinan, China; ^3^Department of Gastroenterology, The 960th Hospital of Chinese PLA Joint Logistics Support Force, Jinan, China

**Keywords:** sarcopenia, NETosis, chemokine, machine learning, nomogram, bioinformatics

## Abstract

**Background:**

Chemokines and neutrophil extracellular trap formation (NETosis) are critical drivers of inflammatory responses. However, the molecular characteristics and interaction mechanisms of these processes in sarcopenia remain incompletely understood.

**Methods:**

Utilizing the mRNA expression profile dataset GSE226151 (including 19 sarcopenia, 19 pre-sarcopenia, and 20 healthy control samples), enrichment analysis was performed to identify differentially expressed NETosis-related genes (DENRGs) and chemokine-related genes (DECRGs). Two machine learning algorithms and univariate analysis were integrated to screen signature genes, which were subsequently used to construct diagnostic nomogram models for sarcopenia. Single-gene Gene Set Enrichment Analysis (GSEA) and Gene Set Variation Analysis (GSVA) were used to investigate pathway associations, followed by the construction of a gene interaction network.

**Results:**

A total of 7 DECRGs and DENRGs were identified, primarily enriched in chemokine signaling pathways, cytokine-cytokine receptor interactions, and sarcopenia-related diseases. Machine learning and univariate analysis revealed three signature genes (CXCR1, CXCR2, and LPL). The nomogram models demonstrated high predictive accuracy in distinguishing sarcopenia from both healthy and pre-sarcopenic states, as evidenced by AUC values of 0.837 (95% CI 0.703–0.947) and 0.903 (95% CI 0.789–0.989), respectively. Single-gene GSEA highlighted significant associations between these genes and the JAK-STAT and PPAR signaling pathways. GSVA indicated that sarcopenia was closely linked to upregulated chemokine signaling, cytokine-receptor interaction activities, and leukocyte transendothelial migration.

**Conclusion:**

The research pinpointed three genes associated with chemokines and NETosis (CXCR1, CXCR2, LPL) and developed highly accurate diagnostic models, offering a new and preliminary approach to differentiate sarcopenia and its early stages.

## Introduction

Sarcopenia is a prevalent age-associated condition characterized by a decline in skeletal muscle mass, muscle strength, and muscle functionality (1). This condition typically exacerbates with obesity and advancing age, emerging as a primary contributor to physical frailty and disability among the elderly population (2, 3). Sarcopenia is caused by chronic inflammation, hormonal changes, malnutrition, abnormal lipid metabolism, and reduced physical activity (4–6). Research shows that sarcopenia negatively affects the quality of life in older adults and is linked to increased falls, fractures, and hospitalizations (7). Those with sarcopenia face more complications than those without (8). With an aging population, the public health impact of sarcopenia is growing. Effective prevention and treatment are needed to improve elderly quality of life and reduce healthcare costs (5, 9). Future research should explore the causes of sarcopenia and develop targeted therapies to maintain muscle health and physical function in older adults (10).

Chronic inflammation plays a key role in the development of sarcopenia. Research shows that ongoing low-grade inflammation is a major factor in this condition (11). In older adults, it is closely linked to declining muscle function and may accelerate muscle loss by affecting protein metabolism and hindering muscle cell regeneration (12). Chronic inflammation is a key factor in sarcopenia, often worsening due to chronic diseases like kidney disease or pancreatitis, which lead to metabolic issues and muscle loss (13, 14). Diet-related inflammation and markers like C-reactive protein (CRP) and systemic immune-inflammatory index (SII) are also linked to sarcopenia, highlighting inflammation’s central role in its development (15, 16). In summary, chronic inflammation is not only a significant characteristic of sarcopenia but also a crucial factor in its pathogenesis (17).

Chemokines and neutrophils are key to inflammation, with chemokines playing a crucial role in recruiting neutrophils during inflammation. They regulate neutrophil release from the bone marrow into the bloodstream and their return for cell death and clearance. Additionally, chemokines influence neutrophil functions like oxidative bursts, degranulation, neutrophil extracellular traps (NETs) formation, and inflammatory mediator production (18). NETs are a defense mechanism used by neutrophils during inflammation to trap and kill pathogens, but they can also cause tissue damage and chronic inflammation (19, 20). Recent studies identify NETs as an effector function of neutrophils, consisting of chromatin networks with histones, myeloperoxidase, and elastase (21, 22). Neutrophils perform their roles through phagocytosis, degranulation, and NET release (23). NETosis is a unique cell death mechanism involving the release of DNA, enzymes, and histones. Activated by chemokines, neutrophils migrate to inflammation sites, produce antimicrobial agents, undergo NETosis, and eliminate bacteria (24, 25).

Chemokines play a crucial role in neutrophil migration and function, with dysfunction potentially linked to sarcopenia. Studies show that CXCL9 is strongly associated with changes in muscle function and higher mortality in older men, indicating chemokines might influence sarcopenia development (26). Furthermore, the increased expression of the chemokine receptor CXCR2 is closely linked to the movement of neutrophils and monocytes, which can affect muscle health if disrupted (27). Neutrophils, macrophages, and T cells are the main cells infiltrating dystrophic muscle (28). Leukocyte infiltration in dystrophic muscle is diverse, including neutrophils, eosinophils, macrophages, and CD8 + and CD4 + T cells (29). Early in muscle repair, immune cells like mast cells and neutrophils clear damaged fibers and release cytokines to recruit macrophages, influencing inflammation (30). Additionally, sarcopenia is linked to chronic inflammation, with neutrophils playing a key role (31). Chemokines and neutrophils likely play key roles in sarcopenia’s onset and progression. Understanding their interactions could lead to new prevention and treatment strategies. However, we need more knowledge about the molecular details of NETosis and chemokines in sarcopenia. Thus, developing a predictive diagnostic model based on these molecular mechanisms is urgently needed.

In modern life sciences research, bioinformatics technology, driven by advancements in high-throughput sequencing and microarray technology, is essential for analyzing gene expression and identifying targets for disease treatment (24). It can extract disease-specific biomarkers from genomic data and reveal molecular mechanisms of diseases, aiding translational medical research. Our study innovatively applied an integrated omics approach. Using sarcopenia-related transcriptomic data from GEO (32), it combined bioinformatics and machine learning to pinpoint NETosis-related genes and chemokine network genes in sarcopenia. Subsequently, a clinically relevant prediction model was created and its diagnostic performance assessed.

## Materials and methods

### Microarray data source information

**FIGURE 1:**
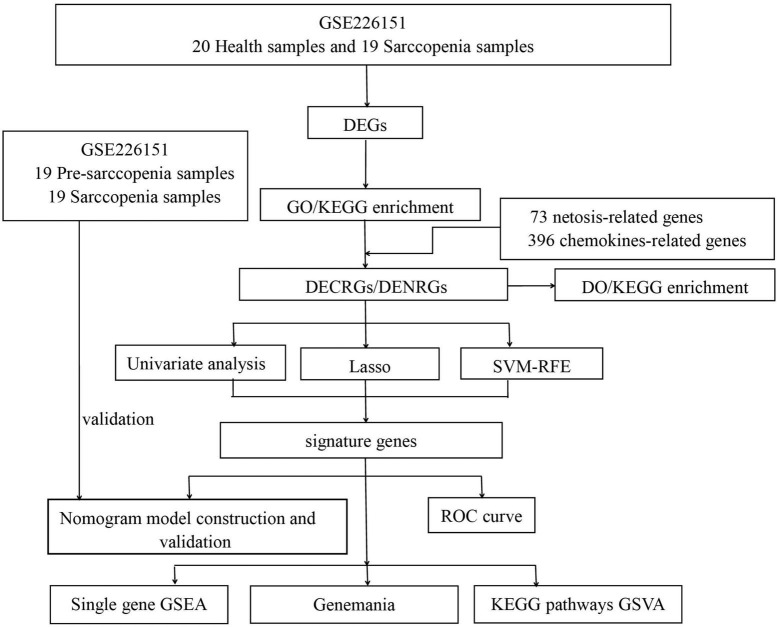
Flowchart of this study. (DEGs, differentially expressed genes; GO, Gene Ontology; KEGG, Kyoto Encyclopedia of Genes and Genomes; DO, Disease Ontology; DECRGs, differentially expressed chemokine-related genes; DENRGs, differentially expressed NETosis-related genes; RF, random forest; SVM-RFE, Support Vector Machines- Recursive Feature Elimination; LASSO, Least Absolute Shrinkage and Selection Operator; GSEA, Gene Set Enrichment Analysis; GSVA, Gene Set Variation Analysis).

The methodology for data analysis employed in this study is illustrated in Figure 1. Inclusion criteria were established to ensure the acquisition of test samples from human subjects, emphasizing independent expression profiles with an adequate sample size. The GSE226151 dataset from the GEO database was selected for inclusion in this investigation. This dataset comprises 19 samples from individuals with sarcopenia, 19 samples from individuals with pre-sarcopenia, and 20 samples from healthy controls, all subjected to mRNA expression profiling. The dataset GSE226151 encompasses healthy controls, individuals with presarcopenia, and those diagnosed with sarcopenia. This extensive inclusion of various stages in the progression of sarcopenia facilitates a more profound understanding of the fundamental mechanisms driving the development of this condition. Due to the larger sample sizes of the sarcopenia and healthy control cohorts, participants were categorized into two groups: a training group, which included samples from sarcopenia and healthy control subjects, and an internal validation group, which consisted of samples from pre-sarcopenia and sarcopenia subjects. Further details are provided in Table 1.

**TABLE 1:** Details of the GEO data.

Dataset	Platform	Number of samples (health/pre-sarccopenia/ sarccopenia)
GSE226151	GPL16791 Illumina HiSeq 2500 (Homo sapiens)	58 (20/19/19)

GEO, Gene Expression Omnibus.

### Discovering genes related to chemokines and NETosis with differential expression

Using the R limma package, we normalized and preprocessed data from sarcopenia patients and healthy controls to identify differentially expressed genes (DEGs). The screening criteria were | log2 fold change| > 0.585 and corrected *p* < 0.05. From the GeneCards database,^1^ we obtained 396 genes associated with chemokines and 73 genes linked to NETosis. The overlap between DEGs and chemokine-associated genes was termed differentially expressed chemokine-related genes (DECRGs). Similarly, In the same manner, we defined genes linked to NETosis that were differentially expressed as DENRGs.

### GO, DO, and KEGG enrichment analyses

Using the R clusterProfiler package, we performed Kyoto Encyclopedia of Genes and Genomes (KEGG) pathway, Disease Ontology (DO), and Gene ontology (GO) enrichment analyses, covering biological process (BP), cellular component (CC), and molecular function (MF). To manage the false discovery rate (FDR), the Benjamini-Hochberg adjustment was applied, with a q-value cutoff of 0.05. The R packages circlize and ggplot2 were used to visualize significant enrichment results.

### Analysis using univariate methods and machine learning techniques like LASSO and SVM-RFE

We performed a univariate analysis on all DECRGs and DENRGs, displaying the results as forest plots using online tools. We then used LASSO regression and SVM-RFE to identify feature genes within these groups. The optimal λ for LASSO was found through 10-fold cross-validation with the R glmnet package, while SVM-RFE was conducted using the R e1071 package. Notably, the penalty parameter (lambda.min) was determined by the minimum criterion for LASSO. We also used the SVM-RFE method to identify key features from input data. A 15-fold cross-validation is applied to test model performance across different feature counts, with error rate and accuracy plots illustrating results. The most significant features are then extracted and saved for further analysis.

The intersection of feature DECRGs, DENRGs, and genes from the univariate analysis was defined as signature genes for further analysis.

### Development of a network of interactions for signature genes

Following this, a network illustrating the interactions among the signature genes was developed using GeneMANIA, an online resource adept at uncovering internal relationships within gene groups.

### Construction of nomogram model and assessment of diagnostic efficacy

Diagnostic models with nomograms were created using signature genes via the R rms package. Calibration was assessed with calibration curves, using mean absolute error and 1,000 bootstrap samples through the R CalibrationCurves package. Decision curve analysis (DCA) evaluated the nomograms’ net benefits at various risk thresholds, and the clinical impact curve (CIC) assessed predictive efficacy. Model performance was analyzed using ROC curves and AUC. A similar nomogram model was developed and validated in an internal validation group.

### Gene set variation analysis and gene set enrichment analysis

This study focuses on exploring the roles of signature genes in sarcopenia by performing single-gene Gene Set Enrichment Analysis (GSEA) using the R clusterProfiler package. Samples were divided into low- and high-expression groups for each gene, and GSEA identified significant KEGG pathways between these groups. Additionally, Gene Set Variation Analysis (GSVA) was conducted using the R GSVA package and KEGG gene sets to compare pathway enrichment. A *p*-value of less than 0.05 was used to determine statistical significance.

## Results

### Recognition of DEGs

**FIGURE 2:**
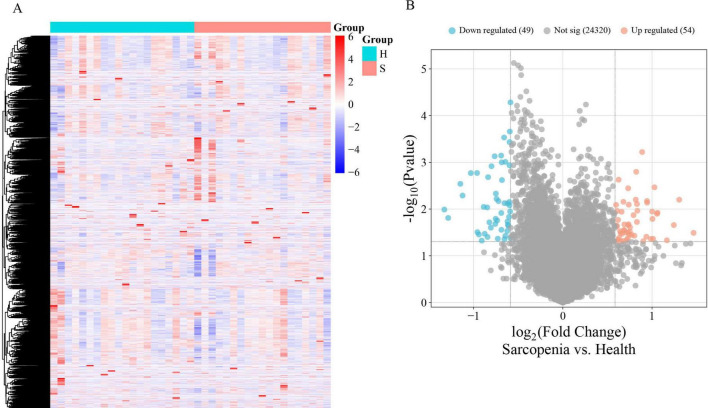
**(A)** The heatmap of DEGs with the two-way clustering; **(B)** The volcano plot of DEGs.

The mRNA expression dataset GSE226151 was normalized, revealing 103 DEGs between sarcopenia and healthy controls, with 54 up-regulated and 49 down-regulated. Figures 2A,B display a heatmap and volcano plot, respectively.

### Function enrichment analyses of the DEGs

**FIGURE 3:**
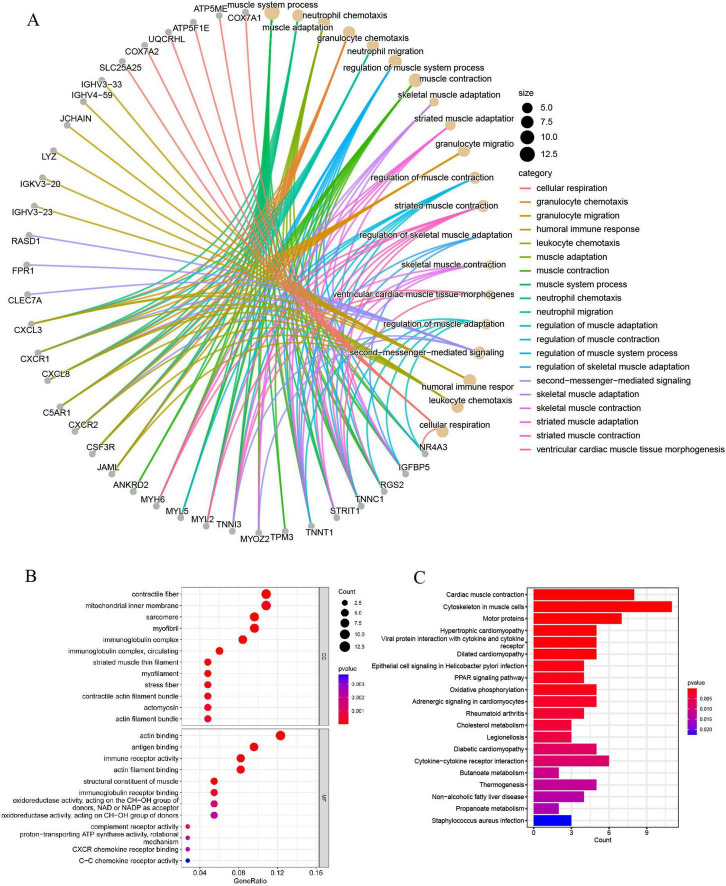
**(A)** The GO BP enrichment analysis of DEGs; **(B)** the GO CC and MF enrichment analysis of DEGs; **(C)** the KEGG pathway enrichment analysis of DEGs.

GO analyses categorized DEGs into BP, CC, and MF, highlighting granulocyte chemotaxis, leukocyte chemotaxis, neutrophil migration, regulation of muscle system processes, and regulation of muscle adaptation as the key processes (Figure 3A). The results indicate that inflammation and neutrophils could be crucial in the development of sarcopenia. Additionally, most DEGs were predominantly localized to muscle-associated tissues and immune complexes, which were significantly involved in processes such as immune receptor activity, immunoglobulin receptor binding, complement receptor activity, CXCR chemokine receptor binding, and C-C chemokine receptor activity (Figure 3B).

KEGG enrichment analysis indicated that DEGs are mainly linked to muscle function, inflammation and chemotaxis, and metabolic regulation. This includes roles in cytoskeletal organization within muscle cells, oxidative phosphorylation, cholesterol metabolism, cytokine-cytokine receptor interactions, non-alcoholic fatty liver disease, diabetic cardiomyopathy, propanoate metabolism, and butanoate metabolism (Figure 3C). In summary, these findings suggest that the functions of neutrophils and chemokines undergo significant alterations in sarcopenia, potentially playing a critical role in its regulation.

### Recognition of DECRGs and DENRGs

**FIGURE 4:**
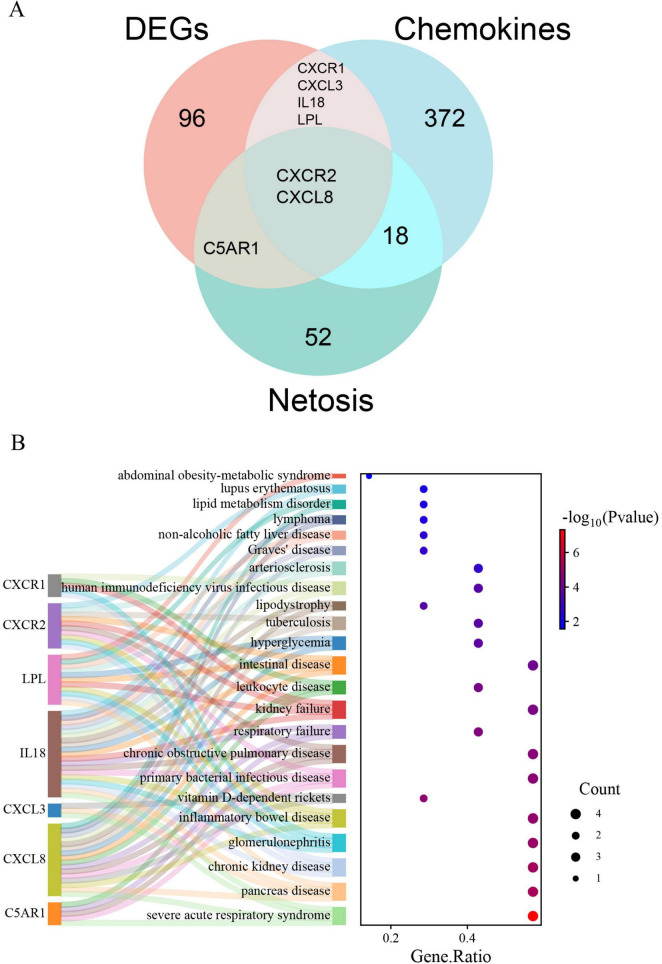
**(A)** Identification of DECRGs and DENRGs between sarcopenia and healthy control groups; **(B)** the DO enrichment analysis of DECRGs and DENRGs.

**FIGURE 5:**
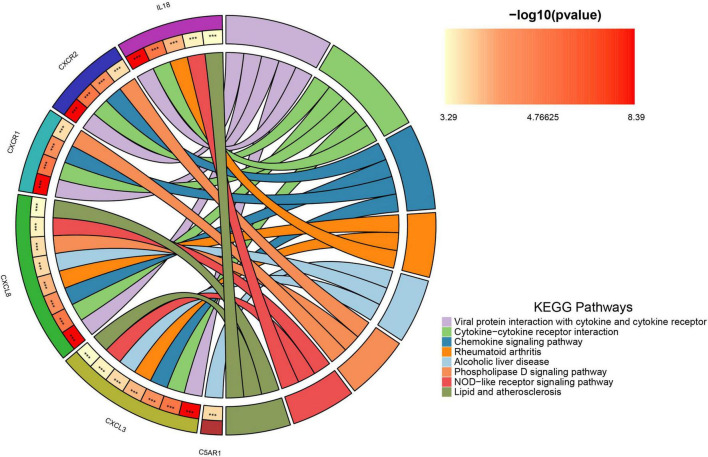
The chordal graph illustrates the significance of KEGG pathway enrichment for DECRGs and DENRGs when comparing sarcopenia to healthy controls, with each string indicating the connection of DECRGs and DENRGs to specific KEGG pathways.

Supplementary materials 1, 2  list 396 chemokine-related and 73 NETosis-related genes. From the GSE226151 dataset, six DECRGs and three DENRGs were identified by intersecting DEGs with chemokine and NETosis-related genes, respectively (Figure 4A). The results of the DO enrichment analyses are depicted in Figure 4B, demonstrating that these seven DECRGs and DENRGs are enriched in several diseases closely associated with sarcopenia, such as abdominal obesity-metabolic syndrome, lipid metabolism disorder, non-alcoholic fatty liver disease, lipodystrophy, tuberculosis, hyperglycemia, leukocyte disease, kidney failure, pancreas disease, vitamin D-dependent rickets, and inflammatory bowel disease. KEGG pathway analysis shows these genes are mainly linked to cytokine-cytokine receptor interaction, chemokine signaling, NOD-like receptor signaling, and viral protein interaction with cytokines, indicating their potential role in immune response regulation and neutrophil chemotaxis (Figure 5).

### Univariate analysis and machine learning algorithm of LASSO and SVM-RFE

**FIGURE 6:**
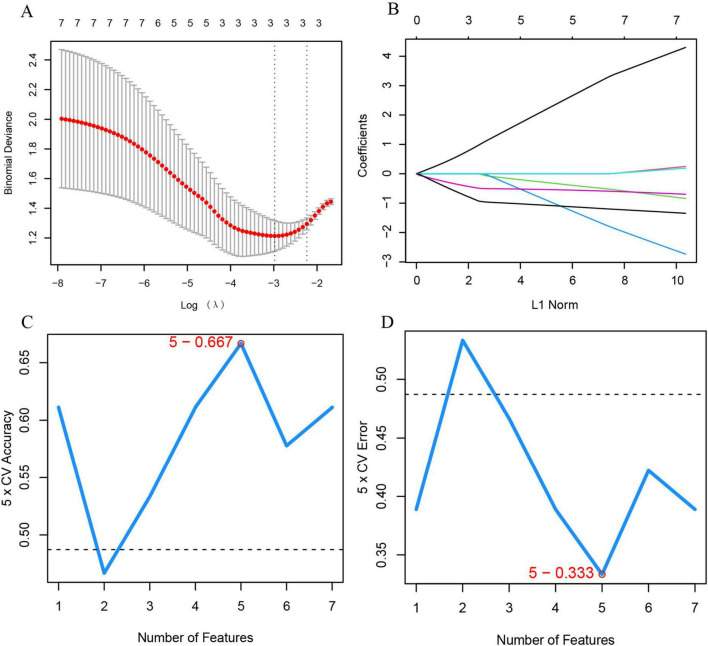
Machine learning for selecting feature genes from DECRGs and DENRGs. **(A)** Optimal log (λ) value is chosen via 10-fold cross-validation and shown by partial likelihood deviance; **(B)** LASSO regression identifies variables and maps them to curves; **(C)** maximum accuracy (5 × CV) of 0.667 is achieved with five feature genes; **(D)** minimum error (5 × CV) of 0.333 occurs with five feature genes.

To identify feature genes within DECRGs and DENRGs, researchers used LASSO and SVM-RFE machine learning algorithms. LASSO regression, which applies L1 regularization for variable selection, employed 10-fold cross-validation to find the most stable model, identifying three feature genes: CXCR1, CXCR2, and LPL (Figures 6A,B). The SVM-RFE method, known for its efficacy in selecting pivotal genes through recursive feature elimination, was subsequently employed. Upon selecting five genes, the model attained optimal performance, evidenced by an accuracy of 66.7% and an error rate of 33.3%. Consequently, the top five genes—CXCR1, CXCR2, CXCL8, IL18, and LPL—were identified from the DECRGs and DENRGs for further analysis (Figures 6C,D).

**FIGURE 7:**
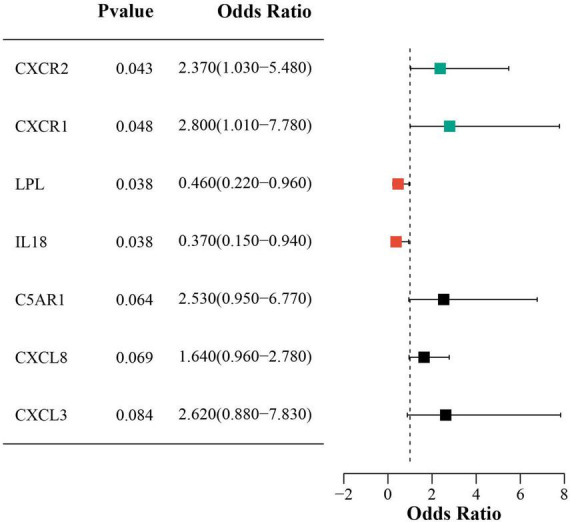
Forest plot of univariate analysis for DECRGs and DENRGs.

Univariate logistic regression showed that higher CXCR2 and CXCR1 expression increased sarcopenia risk, while lower LPL and IL18 expression was protective. Figure 7 presents forest plots that illustrate the risk factors identified through univariate analysis.

**FIGURE 8:**
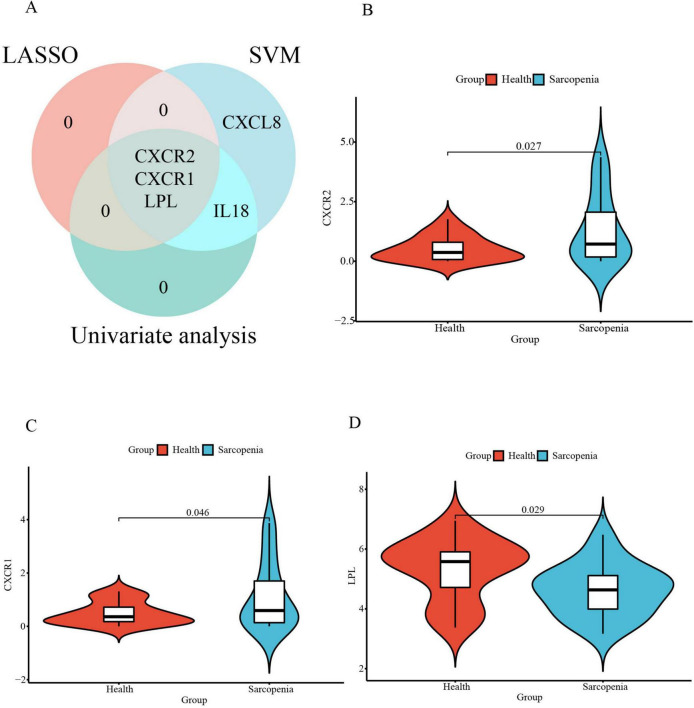
**(A)** The Venn diagram for recognizing signature genes; The violin plot of mRNA expression CXCR2 **(B)**, CXCR1 **(C)**, and LPL **(D)** in GSE226151 between sarcopenia and healthy control groups.

Subsequently, an intersection of these three primary gene sets, as determined by univariate logistic regression, LASSO, and SVM-RFE models, led to the identification of three optimal signature genes—CXCR2, CXCR1, and LPL—for sarcopenia (Figure 8A). Finally, the expression levels of these three signature genes between the sarcopenia and healthy control groups are depicted in the violin plots shown in Figures 8B–D.

### Creation and validation of a predictive model for sarcopenia

**FIGURE 9:**
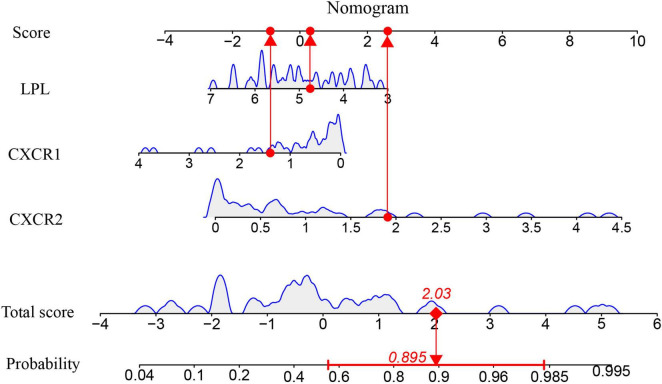
The Nomogram model for predicting sarcopenia from healthy controls based on three signature genes.

**FIGURE 10:**
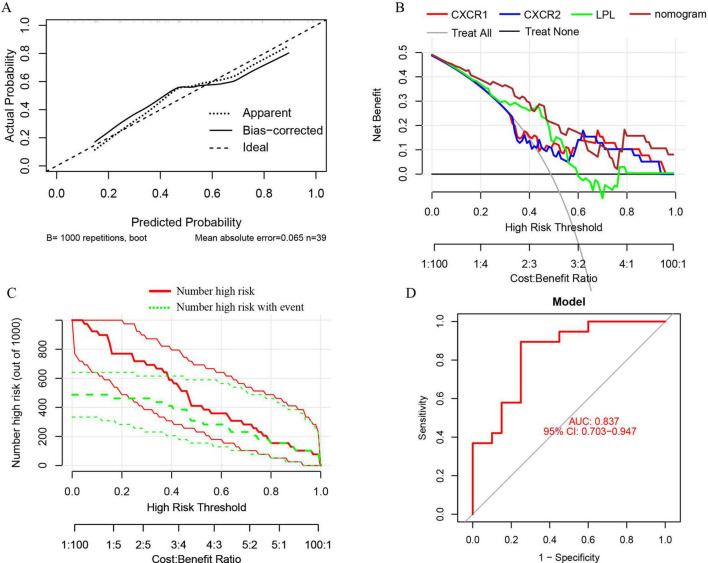
**(A)** Calibration curves for the nomogram with a mean absolute error of 0.065; **(B)** decision curve analysis (DCA) for the nomogram model and each signature gene, comparing “Treat All” and “Treat None” strategies; **(C)** the clinical impact curve (CIC) for the nomogram model; **(D)** ROC and AUC analysis of the nomogram model distinguishing between sarcopenia and healthy control groups.

A nomogram model was developed using logistic regression on three key genes to distinguish sarcopenia patients from healthy individuals, employing the R rms package (Figure 9). A calibration curve showed minimal difference between actual and predicted sarcopenia probabilities, with a mean absolute error of 0.065, indicating high accuracy (Figure 10A). Decision curve analysis (DCA) revealed that this nomogram offered the greatest net benefit for patients (Figure 10B). A clinical impact curve (CIC) based on the DCA curve further demonstrated the model’s clinical effectiveness, with the “Number high risk” curve closely matching the “Number high risk with event” curve at high-risk thresholds of 0.5–1 (Figure 10C).

ROC curves and AUC were used to evaluate the nomogram model’s ability to distinguish sarcopenia patients from healthy controls. The model, incorporating three signature genes, achieved an AUC of 0.837 (95% CI, 0.703–0.947) (Figure 10D), suggesting these genes are crucial in sarcopenia’s pathogenesis.

**FIGURE 11:**
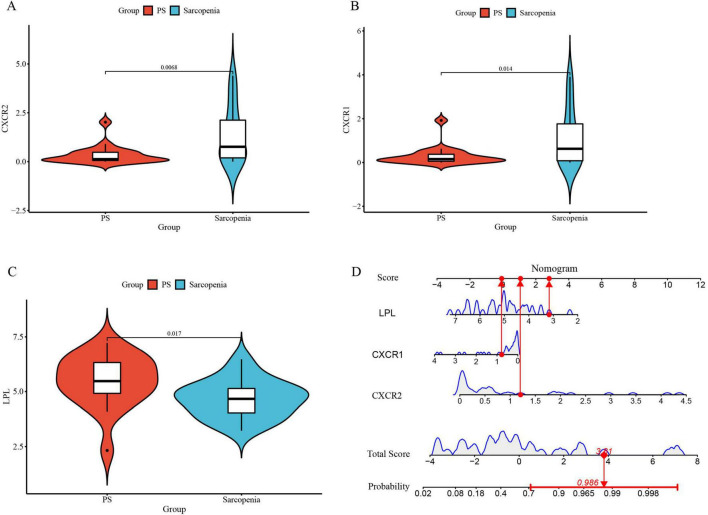
**(A)** The violin plot of mRNA expression CXCR2 between sarcopenia and pre-sarcopenia; **(B)** the violin plot of mRNA expression CXCR1 between sarcopenia and pre-sarcopenia; **(C)** the violin plot of mRNA expression LPL between sarcopenia and pre-sarcopenia; (**D**) the nomogram model for predicting sarcopenia from pre-sarcopenia based on three signature genes (PS, pre-sarcopenia).

To validate these genes and the model’s diagnostic accuracy, we examined their expression in pre-sarcopenia and sarcopenia patients within an internal validation group. CXCR1 and CXCR2 levels were significantly higher, and LPL levels were lower in the sarcopenia group (Figures 11A–C), consistent with the training group. The validation cohort’s nomogram, based on these genes, is shown in Figure 11D.

**FIGURE 12:**
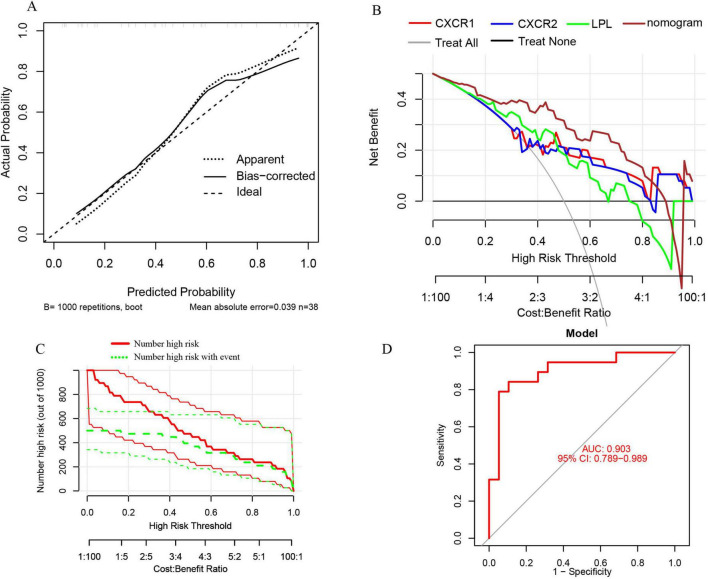
**(A)** Calibration curves for the nomogram with a mean absolute error of 0.039 for predicting sarcopenia from pre-sarcopenia; **(B)** DCA of the nomogram and each signature gene, including “Treat All” and “Treat None” strategies; **(C)** CIC of the nomogram; **(D)** ROC and AUC of the nomogram between sarcopenia and pre-sarcopenia groups.

Furthermore, the nomogram model demonstrates high accuracy, evidenced by a mean absolute error of 0.039 (Figure 12A), as well as substantial net clinical benefit (Figure 12B) and clinical applicability (Figure 12C) in distinguishing sarcopenia from pre-sarcopenia. The AUC for sarcopenia prediction is 0.903 (95% CI 0.789–0.989) (Figure 12D). Collectively, these findings indicate that the three signature genes serve as effective diagnostic indicators for forecasting sarcopenia and differentiating it from pre-sarcopenia.

### Interaction network of signature genes

**FIGURE 13:**
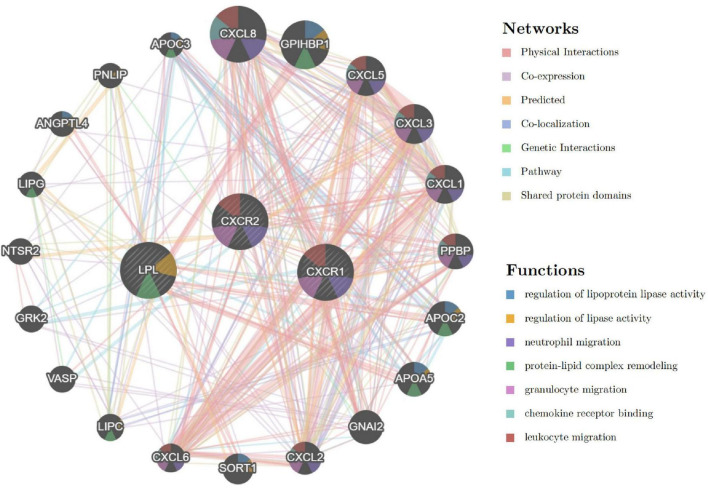
Interaction network of signature genes via GeneMANIA, with line colors indicating gene associations and circle colors representing gene functions.

The analysis of signature genes revealed a complex network involving physical interactions, co-expression, and shared protein domains. Key functions include regulating lipoprotein lipase activity, neutrophil and granulocyte migration, protein-lipid complex remodeling, and chemokine receptor binding (Figure 13 and Supplementary material 3). These findings suggest that chemokine, leukocyte chemotaxis, and lipid metabolism pathways may play a role in sarcopenia’s pathogenesis, highlighting the need for further study.

### Single-gene GSEA and GSVA of signature genes

**FIGURE 14:**
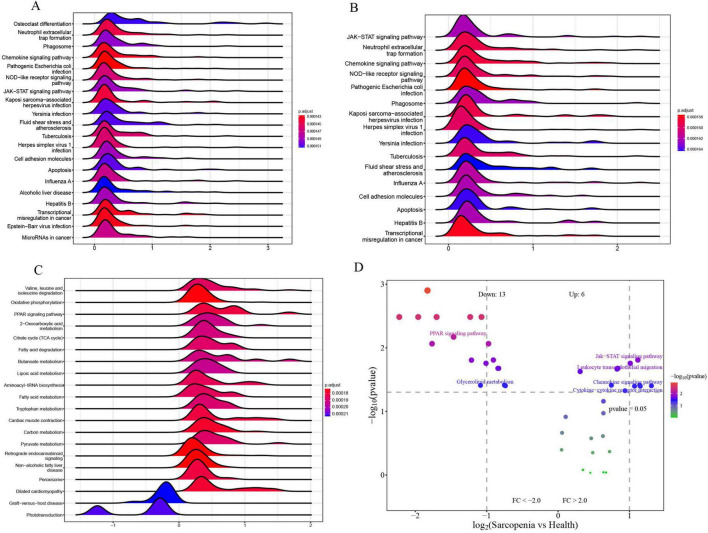
**(A)** Top 20 KEGG pathways from single-gene GSEA of CXCR2; **(B)** top 16 KEGG pathways from single-gene GSEA of CXCR1; **(C)** top 20 KEGG pathways from single-gene GSEA of LPL; **(D)** GSVA-based volcano plot of pathways between sarcopenia and healthy controls.

To explore the roles of CXCR2, CXCR1, and LPL in sarcopenia-related pathways, we analyzed these genes individually using single-gene GSEA and GSVA. Our findings aligned with existing research. By examining mRNA levels, we categorized sarcopenia and healthy samples into high and low expression groups. Single-gene GSEA with KEGG pathways revealed that high CXCR2 expression (Figure 14A) was linked to increased activity in pathways like neutrophil extracellular trap formation, phagosome, chemokine signaling, NOD-like receptor signaling, JAK-STAT signaling, cell adhesion, and apoptosis. Similarly, high CXCR1 expression (Figure 14B) was associated with enhanced activity in neutrophil extracellular trap formation, chemokine signaling, NOD-like receptor signaling, JAK-STAT signaling, and apoptosis.

Moreover, the elevated expression of LPL (Figure 14C) is correlated with enhanced activity in several metabolic and signaling pathways, including oxidative phosphorylation, PPAR signaling, fatty acid degradation, butanoate metabolism, and lipoic acid metabolism. These pathways are identified as potential candidates for further validation.

To identify pathways differently regulated in sarcopenia versus healthy individuals, GSVA analysis was conducted. It showed decreased activity in the PPAR signaling pathway and glycerolipid metabolism, and increased activity in the Jak-STAT signaling pathway, leukocyte migration, chemokine signaling, and cytokine interactions in sarcopenia samples (Figure 14D). Single-gene GSEA confirmed these findings, linking changes in CXCR2, CXCR1, and LPL to the upregulation of Jak-STAT and downregulation of PPAR pathways.

## Discussion

Sarcopenia, an age-related syndrome marked by decreased muscle mass and function, is closely tied to chronic inflammation. Research indicates that this persistent inflammation significantly contributes to sarcopenia by disrupting muscle protein balance, altering metabolism, and causing oxidative stress (33, 34). In the elderly, chronic inflammation, known as “inflammaging,” is a low-grade systemic condition that deteriorates muscle quality and function and is linked to age-related diseases like obesity and cardiovascular issues (35). For instance, studies have demonstrated that chronic inflammation can exacerbate the progression of sarcopenia by influencing muscle metabolism and endocrine functions (36). Chronic inflammation is associated with muscle liposis, especially in liver cirrhosis patients (37). The combination of muscle liposis and sarcopenia significantly increases mortality and hospitalization risks (38). In individuals with chronic kidney disease, chronic inflammation is also a pivotal factor contributing to reduced muscle mass, a condition further aggravated by malnutrition and metabolic acidosis (39, 40). Addressing chronic inflammation may be crucial for preventing and managing sarcopenia. Evidence indicates that anti-inflammatory treatments, nutritional strategies, and proper exercise can improve muscle quality and slow sarcopenia’s progression (34, 40). Future studies should explore how chronic inflammation affects sarcopenia to develop better treatments.

Chronic inflammation, a hallmark of sarcopenia, plays a pivotal role in the condition by contributing to the development of muscle dysfunction (33). Neutrophils are vital to chronic inflammation, serving key roles in the innate immune system and various chronic inflammatory conditions. Recent research shows their diverse functions, extending beyond acute inflammation. They influence immune responses by releasing cytokines and chemokines, persisting in chronic inflammation, and contributing to tissue damage and disease progression (41). Furthermore, the mechanisms of neutrophil death, such as apoptosis and the formation of NETs, are closely associated with the maintenance of chronic inflammation and cancer progression (42). Studies have demonstrated a significant positive correlation between the directional migration of neutrophils and chemokine concentration gradients (43). Chemotactic factors precisely orchestrate neutrophil chemotaxis toward inflammatory foci by establishing molecular concentration gradients (44). In the context of neutrophilic inflammation, activated neutrophils migrate to inflamed regions and participate in inflammatory responses through various mechanisms, including degranulation, oxidative burst, and the formation of NETs (45). The release of NETs is a standard component of the neutrophil response to infection and inflammation (46), and this process is referred to as NETosis (47). Neutrophil-induced NETosis represents a distinct form of cell death, differing from apoptosis and necrosis, and significantly contributes to tissue damage (48, 49). Elevated neutrophil-to-lymphocyte ratio levels may suggest that inflammation plays a substantial role in the development of sarcopenia in the elderly population (33). In response to chemokines, activated neutrophils migrate to the inflammation region, produce antimicrobial agents, and undergo NETosis (25). Consequently, chemokines serve as crucial regulators in the NETosis process. However, The function of chemokine-induced NETosis in the pathways leading to sarcopenia is still uncertain.

Chemokine-regulated NETosis might be crucial in sarcopenia’s development, marked by chronic inflammation. We identified 103 DEGs between sarcopenia patients and healthy controls. GO and KEGG analyses pointed to processes like neutrophil migration and chemokine receptor interactions, supporting our hypothesis and indicating the need for further research.

Additionally, we identified seven DECRGs and DENRGs enriched in the chemokine signaling pathway and chronic inflammatory diseases related to sarcopenia. These diseases include abdominal obesity-metabolic syndrome, lipid metabolism disorder, chronic obstructive pulmonary disease (COPD), kidney failure, inflammatory bowel disease (IBD), and pancreatic disease (50–54). COPD, a prevalent chronic inflammatory lung condition, not only impairs respiratory function but is also associated with systemic inflammation and muscle dysfunction. A decline in muscle strength and mass is commonly observed in COPD patients, and this is closely associated with their persistent inflammatory state (55, 56). Similarly, individuals with kidney failure are susceptible to muscle mass loss and symptoms of sarcopenia due to the accumulation of metabolic waste and persistent inflammation (39, 57). Conditions like Crohn’s disease and ulcerative colitis, which are forms of IBD, are connected to persistent inflammation and sarcopenia. A bidirectional causal relationship may exist between sarcopenia and IBD; IBD can lead to muscle wasting, while sarcopenia may exacerbate the progression of IBD (58). Furthermore, pancreatic disorders, particularly chronic pancreatitis, can contribute to sarcopenia by impairing nutrient absorption and inducing systemic inflammation (13, 59). Sarcopenia is closely associated with chronic inflammatory diseases, worsening muscle quality and function. Variations in genes related to NETosis and chemokines may influence these diseases, leading to sarcopenia. Understanding these connections is crucial for creating effective treatments.

Advancements in big data analytics and AI have enhanced machine learning algorithms for accurate disease classification and prediction (60). A combined LASSO and SVM-RFE algorithm identified three key genes—CXCR2, CXCR1, and LPL—with optimal predictive accuracy. Two validated nomogram models, assessed using calibration curves, DCA, CIC, and ROC curves, showed high efficacy in distinguishing sarcopenia from healthy controls (AUC = 0.837) and pre-sarcopenia (AUC = 0.903). These genes are critically involved in NETosis and chemokine regulation in sarcopenia.

CXCR2 and CXCR1 are key chemokine receptors on neutrophils, crucial for their movement and activation. They are significantly linked to NET formation in inflammation and modulate neutrophil activity in acute respiratory distress syndrome (ARDS) by interacting with chemokines like interleukin-8 (IL-8), promoting lung inflammation and injury (61). In infectious diseases, CXCR2 and CXCR1 are also recognized as key regulators of NET formation. Specifically, in staphylococcus aureus infections, the LukED toxin enhances neutrophil destruction and NET formation by targeting CXCR1 and CXCR2, thus intensifying the infection’s severity (62). Furthermore, evidence shows that CXCR2 is crucial for neutrophil migration and NET formation after peripheral nerve injury, essential for neural regeneration (63). Exploring CXCR1 and CXCR2 expression on muscle cells and their role in muscle inflammation could clarify mechanisms behind sarcopenia. Existing research indicates that CXCR1 and CXCR2 are integral to various cellular processes that could significantly impact muscle health. Notably, these receptors have been implicated in endothelial cell migration and proliferation (64), processes that are vital for muscle repair and regeneration (65). Moreover, research has shown that CXCR1 and CXCR2 play a role in controlling angiogenesis and tissue remodeling (66), both of which are essential for maintaining muscle integrity and function (67). Dysregulation of these processes may contribute to the muscle wasting characteristic of sarcopenia (68, 69). Additionally, the role of CXCR2 and CXCR1 in neutrophil recruitment and activation suggests that they might influence muscle inflammation and subsequent muscle degradation (70, 71). Modulating these receptors could potentially alter the inflammatory environment within muscle tissue, thereby affecting muscle cell survival and function. However, we need to acknowledge that the above analysis is an inference and hypothesis based on the results of previous literature, and there is still a lack of evidence that CXCR1/CXCR2 is directly related to sarcopenia.

Our findings compellingly show that the likelihood of sarcopenia is significantly connected to the heightened expression of CXCR1 and CXCR2, and the KEGG pathway related to “Neutrophil extracellular trap formation.” This observation is consistent with existing literature and supports our hypothesis. In conclusion, the interplay between NETosis, along with its associated chemokine receptors CXCR1 and CXCR2, and sarcopenia encompasses intricate interactions among inflammatory pathways, cellular migration, and tissue remodeling. Gaining more insight into these interactions could lead to new treatment methods focused on minimizing muscle loss and improving muscle function in people with sarcopenia.

Lipoprotein Lipase (LPL) is a crucial enzyme mainly tasked with breaking down triglycerides in the bloodstream, thereby playing a crucial role in lipid metabolism and energy homeostasis (72, 73). The activity of LPL is modulated by a variety of factors, including genetic mutations, protein interactions, and metabolic conditions (74, 75). LPL is not only involved in lipid metabolism but also plays a part in the onset and progression of multiple diseases. For instance, in chronic lymphocytic leukemia, LPL expression correlates with adverse clinical outcomes, although its precise functional roles and regulatory mechanisms remain under investigation (76). Regarding breast cancer, LPL contributes to the energy supply of tumor cells by hydrolyzing lipids from lipoproteins, potentially facilitating tumor growth and progression (77). Furthermore, LPL activity is affected by various pharmacological agents and biomolecules. For instance, metformin is used in diabetes treatment to activate AMP-activated protein kinase (AMPK), which boosts the expression and function of LPL in skeletal muscle, thus enhancing lipid metabolism (78). Actually, tissue remodeling is a significant finding in the study of sarcopenia, characterized by the replacement of healthy muscle with fat and fibrotic tissue (79). The accumulation of excess lipids in skeletal muscles contributes to reduced muscle mass, thereby leading to sarcopenia (80). Myosteatosis, the buildup of fat within muscles, is increasingly noted as a key factor in sarcopenia, especially with the aging population, as it reduces muscle strength and quality (81–84). Decreased LPL activity is closely associated with metabolic syndrome, a cluster of complex metabolic disorders that includes abdominal fat accumulation, elevated triglycerides, high cholesterol, hypertension, hyperglycemia, and non-alcoholic fatty liver disease (85). These elements have been recognized as key contributors to sarcopenia (86). Consequently, the low expression of LPL may be associated with sarcopenia due to disorders of lipid metabolism and myosteatosis. In our study, we observed a significant down-regulation of LPL expression in the sarcopenia group. The elevation in LPL levels was correlated with increased activity in the KEGG pathway terms “Fatty acid degradation” and “Fatty acid metabolism,” as determined through single-gene GSEA. These results are consistent with previous research and may illustrate the significant regulatory role of LPL in the context of sarcopenia.

LPL plays a key role in metabolism and physiological processes like chemokine activity modulation and immune cell chemotaxis. Research shows that miR-467b and miR-590 target LPL in macrophages, reducing lipid accumulation and proinflammatory cytokine secretion (87, 88). Furthermore, angiopoietin-like proteins (ANGPTLs) play a crucial role in regulating LPL activity, which significantly impacts lipid metabolism and the function of immune cells. For instance, ANGPTL4 can inactivate LPL, thereby affecting the hydrolysis of triglycerides and the subsequent release of free fatty acids (89, 90), which are known to modulate inflammatory processes and immune cell behavior (91, 92). LPL is integral to the pathogenesis of atherosclerosis and thrombosis, as evidenced by recent studies (93). Concurrently, chemokines and NETs substantially influence these pathological processes (94, 95). Chemokines, which are tiny signaling proteins, aid in drawing immune cells to inflamed sites, thus significantly influencing the formation and advancement of atherosclerotic plaques. Research indicates that chemokines can augment NET formation by promoting the recruitment and activation of neutrophils, a process intricately linked to the stability of atherosclerotic plaques and the development of thrombosis (96, 97). Therefore, LPL is not only essential for lipid metabolism but also significantly affects immune cell function and chemotaxis through its interactions with chemokines and regulatory proteins. Understanding LPL’s dual role in metabolism and immunity could inform sarcopenia treatments by targeting metabolic and inflammatory pathways. However, research on LPL’s link to sarcopenia via inflammation is scarce, necessitating further studies.

Our research found that CXCR1 and CXCR2 were notably higher in the sarcopenia group, with single-gene GSEA and GSVA confirming their strong association with increased JAK-STAT pathway activity. This pathway is vital for cell proliferation, differentiation, and immune regulation, including the regulation of neutrophil functions. In gastric cancer research, P2RX1 expression in neutrophils is linked to the JAK/STAT signaling pathway. Overexpression of P2RX1 boosts neutrophil survival via this pathway, reducing gastric cancer cell migration, invasion, and proliferation while increasing apoptosis (98). JAK inhibitors can reduce neutrophil activity (99), while cytokines like G-CSF and GM-CSF enhance their survival via the JAK-STAT pathway (100). Additionally, this pathway is crucial in skeletal muscle, where it mediates myokine signaling during contraction and contributes to muscle atrophy in cachexia and chronic kidney disease models (101). Blocking the JAK/STAT pathway pharmacologically in cachectic mice somewhat reduced the loss of muscle mass, as IL-6 activation of this pathway induces muscle atrophy (102, 103).

In the sarcopenia group, LPL perturbation led to down-regulation of the PPAR signaling pathway, which is crucial for lipid and glucose metabolism and inflammatory responses (104). PPARγ and PPARα, key members of this pathway, are essential for adipocyte differentiation and fatty acid oxidation. Overactivation of PPARγ can cause abnormal adipocyte differentiation and lipid accumulation, while PPARα dysfunction may impair fatty acid oxidation, affecting muscle energy metabolism and function (105, 106). Research indicates that regulating the PPAR signaling pathway can enhance mitochondrial biosynthesis and function, impacting muscle energy metabolism and oxidative stress (107, 108). This regulation offers a potential strategy for treating sarcopenia by improving muscle energy metabolism and function (109, 110).

The genes identified in this study, namely CXCR1, CXCR2, and LPL, exhibit substantial potential as clinical diagnostic and therapeutic targets for sarcopenia. Diagnostically, they could form biomarker panels, with their mRNA levels measurable via PCR or RNA sequencing from blood or muscle samples. High CXCR1 and CXCR2 and low LPL levels may indicate sarcopenia progression, aiding early detection. Therapeutically, targeting CXCR1 and CXCR2 with inhibitors or antibodies could reduce inflammation and muscle damage, while enhancing LPL activity with drugs like metformin could improve lipid metabolism and muscle health, potentially preventing muscle lipid accumulation and dysfunction. Future research should prioritize the validation of these targets through clinical trials and the investigation of combination therapies to optimize therapeutic outcomes. Collectively, these genes represent promising avenues for the development of diagnostic tools and interventions aimed at effectively managing sarcopenia.

This study presents several limitations that merit careful consideration. Firstly, the research design was confined to retrospective data analysis and did not include prospective intervention experiments. Furthermore, the GSE226151 dataset employed in this study is characterized by a relatively limited sample size and an absence of baseline data and clinical characteristics of the patients. Additionally, it is lack of external validation data sets. These limitations enhance the potential for selection bias within the study. Secondly, the inflammatory regulatory network is characterized by complex multifactorial interactions, where environmental exposures, genetic backgrounds, and other factors may collectively influence the expression profiles of relevant biomarkers. More critically, the aberrant expression of sarcopenia-related genes was inferred solely from transcriptomic data. Consequently, key scientific questions remain unresolved regarding the dynamic changes at the protein level, their correlations with disease staging and classification, and the specific mechanisms affecting skeletal muscle metabolism. Future research should incorporate proteomic technologies, establish cell models and genetically modified animal models, and validate the biological functions and clinical applications of the target molecules through multicenter, large-sample cohort studies.

However, this study is the first to identify and validate signature genes associated with NETosis and chemokines that can effectively construct a predictive model for sarcopenia. This discovery offers novel insights into the interplay between NETosis, chemokines, and the pathogenesis of sarcopenia, potentially unveiling new therapeutic targets. Importantly, the diagnostic model developed using three signature genes exhibited robust performance in validation group, effectively distinguishing sarcopenia from pre-sarcopenic stages, thereby boosting trust in the dependability of our results. Incorporating these signature genes into current clinical diagnostic systems may be a promising direction for future studies.

## Conclusion

By employing integrative bioinformatics strategies and multiple machine learning algorithms, we have successfully identified three signature chemokine- and NETosis-associated genes (CXCR1, CXCR2, and LPL) and constructed robust diagnostic models for sarcopenia detection. This innovative methodology may represent a preliminary advancement in clinical diagnostics and therapeutic development for sarcopenia management.

## Data Availability

Publicly available datasets were analyzed in this study. This data can be found here: https://www.ncbi.nlm.nih.gov/geo/query/acc.cgi?acc=GSE226151.

## Footnotes

1
https://www.genecards.org/
